# Sleep Quality and Its Association with Quality of Life and Mortality in Hemodialysis Patients

**DOI:** 10.3390/jcm14248729

**Published:** 2025-12-10

**Authors:** Norihito Yoshida, Tatsuki Tanaka, Yusuke Suzuki, Sadamu Takahashi, Mai Hitaka, Shingo Ishii, Keisuke Yamazaki, Yasushi Ohashi

**Affiliations:** Department of Nephrology, Toho University Sakura Medical Center, Chiba 285-8741, Japan; tatsuki.tanaka@med.toho-u.ac.jp (T.T.); yusuke.suzuki@med.toho-u.ac.jp (Y.S.); sadamu.takahashi@med.toho-u.ac.jp (S.T.); mai.hitaka@med.toho-u.ac.jp (M.H.); shingo.ishii@med.toho-u.ac.jp (S.I.); keisuke.yamazaki@med.toho-u.ac.jp (K.Y.); ohashiy@med.toho-u.ac.jp (Y.O.)

**Keywords:** hemodialysis, sleep, quality of life, KDQOL-SF, mortality, prognosis

## Abstract

Sleep disturbances are highly prevalent in hemodialysis (HD) patients and are associated with impaired quality of life (QOL) and poor outcomes. However, the independent impact of sleep quality on QOL and mortality in this population remains unclear. **Methods:** In this multicenter observational study, 346 HD patients completed the Kidney Disease Quality of Life–Short Form (KDQOL-SF). A composite sleep score was derived from sleep-related items, and patients were stratified into tertiles (Q1–Q3). Clinical characteristics, QOL outcomes, and survival were compared across tertiles. Associations between sleep quality and QOL domains were assessed using linear regression, while Cox proportional hazards models were applied to evaluate mortality risk. **Results:** Patients in the lowest tertile (Q1) consistently demonstrated significantly lower scores across multiple SF-36 and kidney-disease-specific QOL domains compared with Q2 and Q3 (all *p* < 0.001). Kaplan–Meier analysis showed lower cumulative survival in Q1 (log-rank *p* = 0.012). In Cox models, Q1 was associated with higher mortality compared to Q3 in both unadjusted (HR, 2.58; 95% CI, 1.46–4.54; *p* = 0.001) and adjusted models (Model 3: HR, 2.04; 95% CI, 1.11–3.77; *p* = 0.023). The associations between Q1 and Q2 were attenuated after full adjustment (HR, 1.88; 95% CI, 0.98–3.60; *p* = 0.058). **Conclusions:** Poor sleep quality was independently associated with impaired QOL and increased all-cause mortality in HD patients. These associations remained significant even after adjustment for inflammation and dialysis adequacy, suggesting that sleep quality reflects a distinct pathophysiological condition and may serve as an important, potentially modifiable indicator of patient well-being and prognosis.

## 1. Introduction

Sleep disturbance is a highly prevalent problem among patients with kidney disease and represents a significant clinical challenge. While the prevalence of insomnia in the general population is estimated to be around 10–30%, it reportedly reaches 30–80% in patients with chronic kidney disease (CKD) [1]. Among patients undergoing hemodialysis (HD), the prevalence is even higher, ranging from 40–85%, likely attributable to the physiological and psychological burdens associated with dialysis treatment [2]. Sleep disturbances are closely linked to comorbidities commonly observed in kidney disease patients, such as diabetes mellitus, hypertension, cardiovascular disease, anemia, depression, and chronic pain [3]. Poor sleep quality leads to deterioration in both physical and mental QOL, decreased daytime activity, increased fatigue, impaired concentration, and a higher risk of developing depression and anxiety [4,5]. These conditions can foster social isolation, creating a vicious cycle of declining QOL [6]. In dialysis patients in particular, lifestyle restrictions due to dialysis schedules, chronic physical discomfort, and persistent fatigue further interact with sleep disturbances, strongly affecting both mental and physical health [7]. Moreover, sleep deprivation can lead to impaired immune function and lower pain thresholds, potentially causing further deterioration in QOL [8]. In addition to its impact on QOL, sleep disturbance is also implicated in patient prognosis, with several studies reporting higher mortality rates among HD patients with poor sleep [9]. Underlying pathophysiological mechanisms—including immune dysfunction, pro-inflammatory responses, and increased cardiovascular risk—are thought to contribute by raising the incidence of infections and cardiovascular events, thereby adversely affecting survival [10,11]. Based on this background, we conducted the present study to clarify the impact of sleep disturbance on QOL and prognosis in HD patients.

## 2. Materials and Methods

### 2.1. Study Design and Participants

From 2019 to 2022, a multicenter, cross-sectional observational study was carried out across four outpatient HD centers operated by the Seijinkai Medical Corporation: Mihama Hospital, Mihama Sakura Clinic, Narita Clinic, and Katori Clinic. The study received ethical approval from the Toho University Sakura Medical Center (Tokyo, Japan; Approval No. S24012_S21073 (S18086)). A total of 1094 patients were undergoing maintenance HD at these facilities during the study period (mean age 68 ± 13 years; 777 men and 317 women). From this population, 428 individuals receiving dialysis during daytime hours were identified via electronic health records and assessed for eligibility. Inclusion criteria were as follows: patients aged 20 years or older who had been on maintenance dialysis for at least 90 days and whose dialysis prescriptions had remained stable for a minimum of 30 days prior to enrollment. The exclusion criteria included recent coronary or valvular surgery, myocardial infarction, unplanned hospitalization for heart failure within the preceding six months, pregnancy, major limb amputation, advanced cancer, dementia, or insufficient clinical data.

### 2.2. Quality of Life Questionnaire

Participants were asked to complete the KDQOL-SF version 1.3 during the first 60 min of their dialysis treatment [12]. This instrument, which is a modified form of the SF-36 health survey, was specifically developed for individuals receiving dialysis and has been validated in Japanese patient populations [13]. The KDQOL-SF includes disease-specific domains that capture both general QOL and issues unique to patients with kidney disease. The instrument consists of ten renal-specific domains: symptom burden, effects on daily living, perceived disease burden, employment status, cognitive function, quality of social relationships, sleep quality, social support, encouragement from dialysis staff, and patient satisfaction. It also incorporates the eight general health domains from the SF-36: physical functioning, role limitations due to physical health problems, bodily pain, general health perceptions, energy/vitality, social functioning, role limitations due to emotional problems, and mental health. A composite sleep score derived from the KDQOL-SF was used as an indicator of sleep quality in the present study. In the Japanese version of the KDQOL-SF, sleep quality is assessed using four questions: overall sleep quality rated on a 10-point scale, the frequency of difficulty returning to sleep after awakening during the night, whether the patient felt they obtained enough sleep, and the frequency of daytime sleepiness. These items were included as the sleep-related components of the questionnaire. The questionnaire was distributed during dialysis sessions, and participants were instructed to complete it either during the session or return it on their next visit. Prior to distribution, dialysis nurses and clinical engineers were briefed on the content and purpose of the survey. These staff members provided patients with the questionnaire, the accompanying explanatory materials, and the consent form, and offered guidance on how to complete the form when needed.

### 2.3. Data Collection

Baseline demographic and clinical information was obtained, including age, sex, diabetes status, dialysis duration, comorbidities, body weight, pre-dialysis blood pressure. Monthly routine blood tests performed for dialysis management were used to gather laboratory data, including blood urea nitrogen (BUN), serum creatinine, calcium, phosphorus, C-reactive protein (CRP), hemoglobin, and intact parathyroid hormone (iPTH) levels. Dialysis adequacy was evaluated using both the urea reduction ratio and the single-pool Kt/V, which was calculated according to the Shinzato formula [14]. Nutritional status was assessed using the Geriatric Nutritional Risk Index (GNRI), calculated with the formula: GNRI = (14.89 + serum albumin [g/dL]) + (41.7 × actual body weight/ideal body weight) [15]. N-terminal pro-brain natriuretic peptide (NT-proBNP) levels prior to dialysis were measured using an electrochemiluminescence immunoassay system (Cobas 8000 e801 module; Roche Diagnostics K.K., Tokyo, Japan).

### 2.4. Statistical Analysis

All statistical analyses were conducted using JMP Pro (version 16.0; SAS Institute Inc., Cary, NC, USA). Responses to the four sleep-related items were converted to a 0–100 scale according to the KDQOL-SF scoring manual, with higher values indicating better sleep. The composite sleep score was calculated as the means of the transformed items. The distribution of these sleep scores was assessed using the Shapiro–Wilk test, which demonstrated a non-normal distribution (W = 0.958, *p* < 0.001). Accordingly, nonparametric statistical methods were applied for all analyses. Participants were stratified into three groups according to tertiles of the sleep score distribution, determined using the 33rd and 66th percentiles: lower tertile (≤52.5), middle tertile (52.6–70), and upper tertile (>70). These percentile-based thresholds were used to compare clinical characteristics, QOL scores, and survival outcomes across groups. Continuous variables, including laboratory findings and QOL scores, were reported as medians with interquartile ranges (IQRs). Intergroup comparisons of continuous data were conducted using the Kruskal–Wallis’s test, appropriate for evaluating non-normally distributed variables across more than two groups. Categorical variables were summarized as counts and percentages and compared using Pearson’s chi-square test. For survival analysis, Kaplan–Meier curves were generated to compare cumulative survival among the three sleep quality groups. The log-rank test was used to assess statistical significance. A *p*-value < 0.05 was considered statistically significant in all analyses.

Use of Generative AI: Generative artificial intelligence (ChatGPT5.1, OpenAI, San Francisco, CA, USA) was used only for minor language editing and formatting of the manuscript. The study design, data analysis, and interpretation were performed independently by the authors.

## 3. Results

### 3.1. Baseline Characteristics

The clinical and laboratory characteristics of patients stratified by sleep score tertiles are summarized in Table 1. No significant differences were observed among the three groups with respect to age, sex distribution, BMI, dialysis vintage, or major comorbidities. Most laboratory parameters, including serum albumin, electrolytes, hemoglobin, and inflammatory markers, also did not differ across groups. However, Kt/V was significantly higher in the highest tertile (Q3) compared with the lowest tertile (Q1) (*p* = 0.034).

### 3.2. QOL Scores According to Sleep Score Tertiles

**Figure 1:**
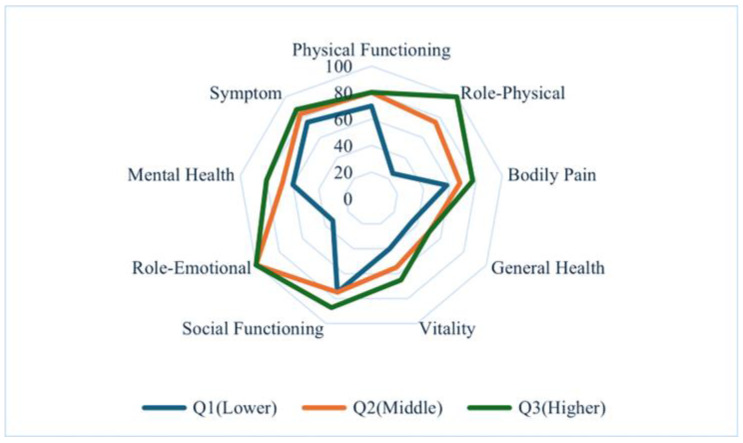
Radar Chart of SF-36 Domains Across Sleep Score Tertiles. Note: Median scores for eight SF-36 domains across sleep-score tertiles. Higher values indicate better health-related QOL.

**Figure 2:**
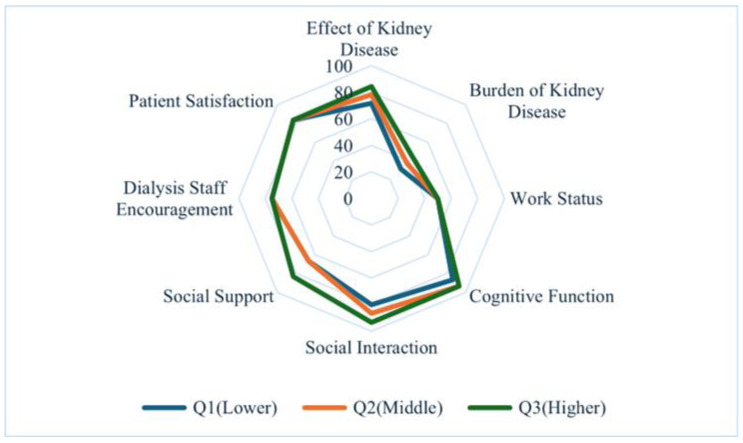
Radar Chart of KDQOL-Specific Domains Across Sleep Score Tertiles. Note: Median scores for kidney-disease-specific domains. Group definitions as in Figure 1; higher values denote better perceived outcomes.

Comparisons of health-related QOL scores are presented in Table 2 and Figure 1 and Figure 2. Patients in the lowest tertile (Q1) had significantly lower scores across most domains of the SF-36 and KDQOL-SF compared with those in middle tertile (Q2) and highest tertile (Q3) (all *p* < 0.001). The only exception was work status, which did not differ significantly among the three groups (*p* = 0.281).

### 3.3. Multiple Analysis

**Figure 3:**
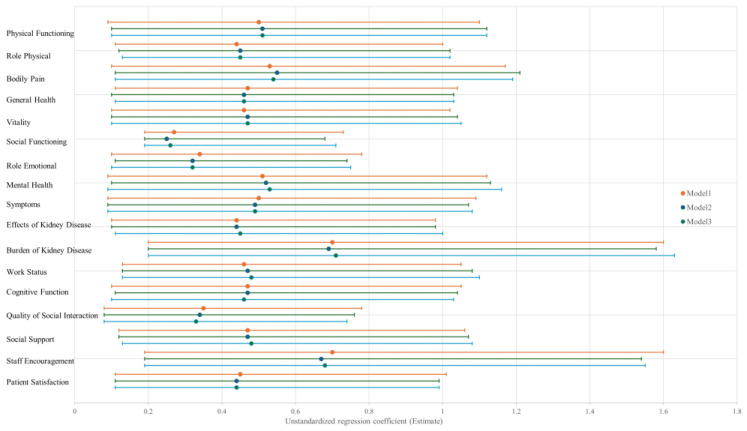
Association between sleep score and QOL domains across multivariable models. Note: Each point represents the regression coefficient (±95% CI) for a one-unit increase in the sleep score across Models 1–3 (defined in Table 3).

Table 3 and Figure 3 present the results of stepwise multivariable regression analyses examining the association between sleep score and each QOL domain. In the unadjusted model (Model 1), the sleep score was significantly and positively associated with all 17 QOL domains, including both SF-36 and kidney-disease-specific subscales. These associations remained consistent in the fully adjusted model (Model 3), which accounted for potential confounders such as age, sex, Kt/V, diabetes mellitus, BMI, GNRI, CVD, NT-proBNP, and CRP. In Model 3, the strongest standardized associations were observed in the Symptoms/Problems (standardized β = 0.50, *p* < 0.001), Effect of Kidney Disease (β = 0.51, *p* < 0.001), Patient Satisfaction (β = 0.50, *p* < 0.001), Cognitive Function (β = 0.45, *p* < 0.001), and Vitality (β = 0.42, *p* < 0.001) domains. Furthermore, Physical Functioning, Role-Physical, Bodily Pain, and Mental Health also demonstrated robust associations, with standardized β values ranging from 0.35 to 0.42, all with *p* < 0.001. Although most QOL domains showed highly significant associations, Work Status was the only domain with a *p* value exceeding the threshold of 0.001 (β = 0.26, 95% CI: [0.07, 0.45], *p* = 0.006), suggesting a comparatively weaker yet still statistically significant relationship. The observed consistency across models reinforces the independent impact of sleep score on multiple dimensions of QOL in patients undergoing maintenance HD. Figure 3 visually summarizes these findings. For each QOL domain, the unstandardized regression coefficients and 95% confidence intervals are displayed for Models 1 through 3. All intervals in Model 3 excluded the null value, further supporting the robustness of the associations.

### 3.4. Mortality Analysis

**Figure 4:**
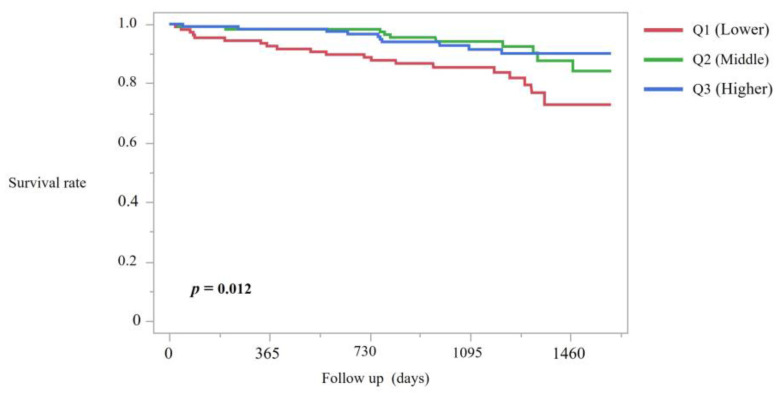
Kaplan–Meier Curves for All-Cause Mortality Stratified by Sleep Score Tertiles. Note: Kaplan–Meier curves show cumulative survival across sleep score tertiles. Survival differences were evaluated using the log-rank test.

Among 346 patients included in the survival analysis, a total of 40 deaths occurred during a median follow-up period of approximately 3.8 years. Kaplan–Meier analysis demonstrated significant differences in cumulative survival among sleep score tertiles (log-rank χ^2^ = 8.83, *p* = 0.012; Figure 4). The lowest tertile (Q1) showed 20 deaths among 109 patients, compared with 10 deaths in each of the middle tertile (Q2) and highest tertile (Q3). The estimated mean survival times (±SE) were 1218.2 ± 35.4 days in Q1, 1408.4 ± 22.2 days in Q2, and 1165.1 ± 16.6 days in Q3. Median survival times could not be precisely calculated due to censoring; however, the estimated 25th percentile survival time in Q1 was 1365 days.

### 3.5. Multiple Analysis of Mortality

**Figure 5:**
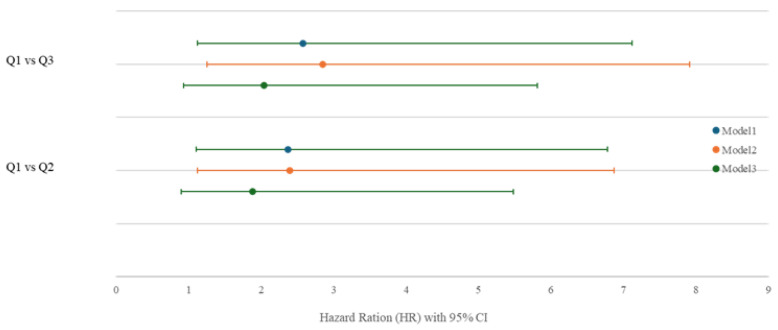
Hazard Ratios for Mortality by Sleep Quality (Q1 vs. Q3 and Q2). Note: Hazard ratios (95% CI) for mortality across sleep tertiles, derived from Cox proportional hazards models (Models 1–3).

To further examine the relationship between sleep quality and mortality risk, stepwise Cox proportional hazards models were constructed (Table 4 and Figure 5). The highest tertile (Q3) served as the reference category. In the unadjusted model (Model 1), patients in the lowest tertile (Q1) exhibited a significantly higher mortality risk compared with those in Q3 (HR, 2.58; 95% CI, 1.46–4.54; *p* = 0.001). This association persisted after adjustment for age, sex, and Kt/V (Model 2: HR, 2.85; 95% CI, 1.60–5.06; *p* < 0.001). In the fully adjusted model (Model 3), which additionally controlled for DM, CVD, BMI, NT-proBNP, CRP, and GNRI, the association remained significant though attenuated (HR, 2.04; 95% CI, 1.11–3.77; *p* = 0.023). When Q1 was compared with the middle tertile (Q2), higher mortality risk was observed in Q1 in both the unadjusted and partially adjusted models (Model 1: HR, 2.37; 95% CI, 1.27–4.41; *p* = 0.006; Model 2: HR, 2.39; 95% CI, 1.27–4.48; *p* = 0.007). However, in the fully adjusted model, the association was attenuated and no longer statistically significant (HR, 1.88; 95% CI, 0.98–3.60; *p* = 0.058).

## 4. Discussion

As mentioned in the introduction, sleep disturbance is highly prevalent among HD patients and constitutes a major clinical concern because of its association with impaired QOL and adverse prognosis [1,2,9]. The present study was designed to clarify the relationships between sleep quality, QOL, and survival in this population. The principal finding was that lower KDQOL-SF-derived sleep scores were independently associated with worse outcomes across multiple QOL domains and with higher all-cause mortality. Both Kaplan–Meier analysis and Cox proportional hazards models consistently demonstrated that patients in the lowest sleep tertile (Q1) had significantly higher mortality than those in the highest tertile (Q3), even after adjustment for demographic, nutritional, and clinical variables, suggesting that patient-reported sleep quality functions as an independent prognostic indicator rather than merely a correlate of comorbidity [16].

By evaluating QOL and mortality within the same cohort, our study provides an integrated perspective on how sleep relates to psychosocial well-being and survival. Because baseline characteristics such as age, comorbidities, and BMI were not significantly imbalanced across tertiles, the differences in outcomes are unlikely to be explained solely by these factors. The robust associations observed across numerous SF-36 and kidney-disease-specific domains indicate that poor sleep exerts broad effects on physical and mental health, including fatigue, reduced daytime activity, cognitive difficulties, and mood symptoms [4,5,6,17]. Psychosocial domains—such as social support, treatment satisfaction, and quality of social interactions—were also linked to sleep quality, implying potential bidirectional interactions between sleep and psychosocial well-being [18,19]. By contrast, associations with work status and the burden of kidney disease were comparatively weaker, which may reflect the influence of structural or socioeconomic determinants that can overshadow direct effects of sleep on daily functioning [3,7].

The mechanisms that may connect poor sleep with adverse outcomes are likely multifactorial. Sympathetic overactivity, immune dysregulation, sustained inflammation, and cardiovascular stress have all been implicated and could plausibly increase the risks of hypertension, arrhythmia, infection, and cardiovascular events in HD patients [9,10,11,20]. In our cohort, CRP showed a marginal association with sleep score, consistent with prior observations that heightened inflammatory activity is related to poorer sleep [21]. Elevated inflammatory markers have also been associated with lower QOL and increased mortality in both dialysis and general populations [22,23]. Importantly, the association between the lowest sleep tertile and mortality persisted after adjustment for CRP, suggesting that sleep influences prognosis through pathways extending beyond systemic inflammation. Dialysis adequacy, reflected by Kt/V, was significantly associated with sleep at baseline, raising the possibility that better toxin clearance and fluid management may ameliorate uremia-related symptoms—such as pruritus, cramps, and restless legs syndrome—that impair sleep [24,25]. Nonetheless, the mortality association between Q1 and Q3 remained after adjustment for Kt/V, indicating that sleep quality likely represents a distinct prognostic construct.

Taken together, these findings support the view that poor sleep quality in HD patients reflects a multifactorial pathophysiological state characterized by autonomic dysregulation, oxidative stress, and circadian disruption, which may heighten cardiovascular and infectious vulnerability [26]. From a clinical standpoint, routine assessment of sleep using a brief patient-reported measure, such as the KDQOL-SF–derived sleep score employed here, could help identify individuals at increased risk of diminished well-being and adverse outcomes without requiring additional testing. Because sleep disturbance is potentially modifiable, targeted strategies—including optimization of dialysis prescriptions and fluid balance, behavioral therapy, and carefully selected pharmacologic approaches—may yield meaningful improvements in QOL and possibly survival, echoing recommendations from the broader sleep literature [27]. Future work should test whether structured sleep interventions can causally improve clinical endpoints in HD populations and should incorporate objective assessments (e.g., actigraphy or polysomnography) to delineate the contributions of specific disorders such as insomnia, sleep apnea, and restless legs syndrome [8,16,20].

### Limitation

This study has several limitations that should be acknowledged.

First, QOL and sleep quality were evaluated using the self-administered KDQOL-SF questionnaire. As a subjective assessment, it is prone to perception and reporting bias, and the absence of objective sleep measures—such as polysomnography or actigraphy—limits the precision of the results.

Second, information regarding the use of hypnotic agents or other sleep medications was not collected, preventing evaluation of their potential influence on sleep and related outcomes.

Third, psychiatric conditions such as depression, anxiety, and cognitive impairment were not comprehensively assessed. These factors could have acted as residual confounders, particularly for QOL domains related to mental health.

Fourth, the present study examined overall sleep disturbance but did not distinguish among specific types, such as insomnia, sleep apnea, or restless legs syndrome, each of which may have unique effects on QOL and prognosis.

Fifth, sleep scores were divided into tertiles based on the distribution within this cohort rather than clinically validated thresholds, which may limit generalizability.

Sixth, because the analyses were largely cross-sectional, causal relationships between sleep quality, QOL, and prognosis cannot be established. Although survival was examined retrospectively, these associations should be interpreted as correlations rather than evidence of causality.

Seventh, causes of death were not systematically classified, preventing determination of whether the excess mortality observed in the lowest sleep score group was primarily due to cardiovascular, infectious, or other events.

Eighth, although data were collected from multiple facilities, all centers belonged to a single medical corporation within one geographic region, which may introduce institutional or regional bias. The moderate sample size also limits statistical power and external validity.

Finally, as with all observational studies, residual confounding from unmeasured factors cannot be excluded.

## 5. Conclusions

In this multicenter study of patients receiving maintenance HD, lower KDQOL-SF sleep scores—indicating poorer sleep quality—were independently associated with impaired QOL across multiple domains and with an increased risk of all-cause mortality. These associations remained significant after adjustment for established clinical risk factors, suggesting that sleep quality may serve as an important indicator of patient well-being and prognosis.

## Figures and Tables

**Table 1:** Baseline Characteristics of HD Patients Stratified by Sleep Score.

Item	Q1 Median (IQR)	Q2 Median (IQR)	Q3 Median (IQR)	*p* Value
Age (years)	67 (52–74)	66 (56–73)	67 (58–73)	0.559
BMI (kg/m^2^)	22.11 (19.60–25.12)	22.15 (19.96–25.85)	22.04 (19.51–24.98)	0.617
HD history (months)	66 (27.50–126)	74 (36.50–128.5)	67 (37–138.5)	0.840
Initial SBP (mmHg)	148 (130–162)	144.5 (128–164.75)	146 (130–159.5)	0.834
Initial DBP (mmHg)	76 (68–87)	74 (66–87.5)	78 (68–85)	0.857
TP(g/dL)	6.5 (6.3–6.9)	6.6 (6.2–6.8)	6.5 (6.2–6.8)	0.973
Alb (g/dL)	3.7 (3.4–3.8)	3.6 (3.4–3.8)	3.6 (3.45–3.8)	0.697
Na (mEq/L)	139 (137–141)	139 (138–141)	139 (138–140)	0.786
K (mEq/L)	4.8 (4.3–5.25)	4.8 (4.3–5.275)	4.8 (4.45–5.4)	0.453
Cl (mEq/L)	103 (102–105)	103 (101–106)	103 (102–105)	0.688
TG (mg/dL)	97 (67.5–145.5)	108 (75–151)	103 (68–152.5)	0.856
LDL-C (mg/dL)	87 (70–105)	85.5 (66–103.75)	85 (71–110)	0.449
UA (mg/dL)	7.8 (7–8.7)	7.8 (6.9–8.4)	7.5 (6.65–8.45)	0.060
BUN (mg/dL)	58.6 (49.2–67.05)	57.35 (48.025–72.025)	55.2 (45.85–66.35)	0.433
Cr (mg/dL)	10.13 (8.63–12.09)	10.455 (8.9825–11.76)	10.16 (8.855–11.445)	0.705
Ca (mg/dL)	8.7 (8.2–9)	8.7 (8.3–9)	8.6 (8.3–8.9)	0.477
P (mg/dL)	5.6 (5.1–6.3)	5.6 (4.8–6.275)	5.4 (4.7–6.2)	0.303
HbA1c (%)	6.25 (5.7–6.875)	6.2 (5.6–6.8)	6.3 (5.6–6.775)	0.753
iPTH (pg/mL)	174 (109–222)	134.5 (87.25–205.75)	158 (113.5–210.5)	0.315
β2MG (mg/L)	26.6 (22.55–29.75)	26.8 (23.95–29.55)	26 (23.3–29.25)	0.63
CRP (mg/dL)	0.13 (0.05–0.296)	0.1135 (0.04525–0.3525)	0.08 (0.04–0.2035)	0.060
Hb (g/dL)	11.2 (10.5–12)	11.3 (10.7–11.8)	11.3 (10.725–11.975)	0.956
Plasma Water Index	2.64 (1.41–3.40)	2.42 (1.53–3.30)	2.39 (1.69–3.15)	0.565
KT/Vurea (D)	1.74 (1.60–1.95)	1.81 (1.65–2.07)	1.88 (1.68–2.12)	0.034
NT-proBNP (pg/mL)	3330 (1365–8175)	3940 (1945–8870)	3140 (1905–6615)	0.541
GNRI	97.23 (90.06–103.67)	96.34 (89.69–103.29)	95.92 (89.59–101.82)	0.90

Notes: Data are presented as median (interquartile range) or number (percentage). Patients were stratified into tertiles based on the KDQOL-SF–derived sleep score (Q1, lowest; Q3, highest). *p* values were calculated using the Kruskal–Wallis’s test for continuous variables and Pearson’s χ^2^ test for categorical variables.

**Table 2:** Health-related quality of life scores across sleep score tertiles.

Test Item	Q1 Median (IQR)	Q2 Median (IQR)	Q3 Median (IQR)	*p* Value
Physical Functioning	70 (40–80)	80 (65–95)	80 (65–95)	<0.001
Role-Physical	25 (0–100)	75 (0–100)	100 (50–100)	<0.001
Bodily Pain	57.5 (32.5–87.5)	67.5 (53.125–90)	77.5 (55–100)	<0.001
General Health	35 (22.5–50)	50 (35–50)	50 (40–60)	<0.001
Vitality	40 (25–57.5)	55 (45–70)	65 (45–80)	<0.001
Social Functioning	75 (37.5–87.5)	75 (62.5–100)	87.5 (62.5–100)	<0.001
Role-Emotional	33.33 (0–100)	100 (0–100)	100 (33.3–100)	<0.001
Mental Health	60 (42–76)	68 (56–84)	80 (60–88)	<0.001
Symptom	75 (59.4–85.4)	83.3 (77.1–91.7)	87.5 (79.7–94.8)	<0.001
Effect of Kidney Disease	71.9 (48.4–84.5)	78.1 (66.4–87.5)	84.4 (75–93.8)	<0.001
Burden of Kidney Disease	31.3 (6.25–46.9)	37.5 (25–50)	43.75 (31.3–56.3)	<0.001
Work Status	50 (0–100)	50 (0–100)	50 (0–100)	0.281
Cognitive Function	86.7 (66.7–100)	93.3 (80–100)	93.3 (86.7–100)	<0.001
Social Interaction	80 (53.3–100)	86.7 (73.3–100)	93.3 (80–100)	<0.001
Social Support	66.7 (50–83.3)	66.7 (66.7–83.3)	83.3 (66.7–100)	<0.001
Dialysis Staff Encouragement	75 (50–87.5)	75 (50–87.5)	75 (62.5–100)	<0.001
Patient Satisfaction	83.3 (66.7–100)	83.3 (66.7–100)	83.3 (83.3–100)	<0.001

Notes: Values are median (interquartile range). Group definitions are as in Table 1. Higher scores indicate better QOL. *p* values were derived from the Kruskal–Wallis’s test.

**Table 3:** Association between Sleep KD and QOL Domains: Comparison Across Stepwise Adjusted Models.

QOL Domain	Model 1 (Unadjusted) [95% CI] (Std β)	*p* Value	Model 2 (Adjusted) [95% CI] (Std β)	*p* Value	Model 3 (Adjusted) [95% CI] (Std β)	*p* Value
Physical Functioning	0.45 [0.34, 0.56] (0.40)	<0.001	0.44 [0.33, 0.55] (0.39)	<0.001	0.44 [0.33, 0.55] (0.39)	<0.001
Role-Physical	0.70 [0.51, 0.90] (0.36)	<0.001	0.67 [0.48, 0.87] (0.34)	<0.001	0.68 [0.49, 0.87] (0.35)	<0.001
Bodily Pain	0.47 [0.35, 0.59] (0.38)	<0.001	0.47 [0.35, 0.60] (0.38)	<0.001	0.48 [0.35, 0.60] (0.38)	<0.001
General Health	0.35 [0.27, 0.43] (0.42)	<0.001	0.34 [0.26, 0.42] (0.40)	<0.001	0.33 [0.25, 0.41] (0.40)	<0.001
Vitality	0.47 [0.37, 0.58] (0.43)	<0.001	0.47 [0.36, 0.57] (0.43)	<0.001	0.46 [0.36, 0.57] (0.42)	<0.001
Social Functioning	0.46 [0.33, 0.59] (0.35)	<0.001	0.47 [0.34, 0.61] (0.36)	<0.001	0.48 [0.35, 0.62] (0.37)	<0.001
Role-Emotional	0.70 [0.50, 0.90] (0.34)	<0.001	0.69 [0.49, 0.89] (0.34)	<0.001	0.71 [0.51, 0.92] (0.35)	<0.001
Mental Health	0.44 [0.34, 0.54] (0.41)	<0.001	0.44 [0.34, 0.54] (0.41)	<0.001	0.45 [0.34, 0.55] (0.42)	<0.001
Symptom	0.50 [0.41, 0.59] (0.51)	<0.001	0.49 [0.40, 0.58] (0.50)	<0.001	0.49 [0.40, 0.59] (0.50)	<0.001
Effect of Kidney Disease on Daily Life	0.51 [0.42, 0.61] (0.50)	<0.001	0.52 [0.42, 0.61] (0.50)	<0.001	0.53 [0.44, 0.63] (0.51)	<0.001
Burden of Kidney Disease	0.34 [0.24, 0.44] (0.33)	<0.001	0.32 [0.21, 0.42] (0.30)	<0.001	0.32 [0.22, 0.43] (0.31)	<0.001
Work Status	0.27 [0.08, 0.46] (0.15)	0.006	0.25 [0.06, 0.43] (0.14)	0.010	0.26 [0.07, 0.45] (0.14)	0.006
Cognitive Function	0.46 [0.36, 0.56] (0.44)	<0.001	0.47 [0.37, 0.57] (0.44)	<0.001	0.47 [0.37, 0.58] (0.45)	<0.001
Quality of Social Interaction	0.47 [0.36, 0.57] (0.43)	<0.001	0.46 [0.36, 0.57] (0.42)	<0.001	0.46 [0.35, 0.57] (0.42)	<0.001
Social Support	0.53 [0.43, 0.64] (0.46)	<0.001	0.55 [0.44, 0.66] (0.47)	<0.001	0.54 [0.43, 0.65] (0.47)	<0.001
Dialysis Staff Encouragement	0.44 [0.33, 0.56] (0.37)	<0.001	0.45 [0.33, 0.57] (0.38)	<0.001	0.45 [0.32, 0.57] (0.37)	<0.001
Patient Satisfaction with Dialysis Care	0.50 [0.41, 0.60] (0.49)	<0.001	0.51 [0.41, 0.61] (0.50)	<0.001	0.51 [0.41, 0.61] (0.50)	<0.001

Notes: Values represent unstandardized regression coefficients (95% CI) and standardized β. Model 1 was unadjusted; Model 2 adjusted for age, sex, and Kt/V; Model 3 further adjusted for diabetes, BMI, GNRI, CVD, NT-proBNP, and CRP. *p* values < 0.001 were considered significant.

**Table 4:** Hazard Ratios and 95% Confidence Intervals for Mortality according to Sleep Score Groups in Stepwise Adjusted Cox Proportional Hazards Models

Comparison	Model 1 > HR (95% CI), *p* Value	Model 2 > HR (95% CI), *p* Value	Model 3 > HR (95% CI), *p* Value
**Q1 vs. Q3**	2.58 (1.46–4.54), 0.001	2.85 (1.60–5.06), <0.001	2.04 (1.11–3.77), 0.023
**Q1 vs. Q2**	2.37 (1.27–4.41), 0.006	2.39 (1.27–4.48), 0.007	1.88 (0.98–3.60), 0.058

Notes: Hazard ratios (95% CI) for all-cause mortality across sleep tertiles estimated by stepwise Cox models (Models 1–3).

## Data Availability

The datasets generated and analyzed during the current study are available from the corresponding author on reasonable request.

## Abbreviations

The following abbreviations are used in this manuscript:

Alb	Albumin
AUC	Area under the curve
BIA	Bioelectrical impedance analysis
BUN	Blood urea nitrogen
BMI	Body mass index
Ca	Calcium
CI	Confidence interval
Cl	Chloride
Cr	Creatinine
CRP	C-reactive protein
CVD	Cardiovascular disease
DM	Diabetes mellitus
DBP	Diastolic blood pressure
ECW/ICW	Extracellular water to intracellular water ratio
GNRI	Geriatric nutritional risk index
Hb	Hemoglobin
HD	Hemodialysis
HDL-C	High-density lipoprotein cholesterol
HR	Hazard ratio
iPTH	Intact parathyroid hormone
Kt/V	Urea clearance normalized by distribution volume
K	Potassium;
Na	Sodium
NT-proBNP	N-terminal pro-brain natriuretic peptide
P	Phosphate
PA	Phase angle
PEW	Protein-energy wasting
QOL	Quality of life
ROC	Receiver operating characteristic
SBP	Systolic blood pressure
SF-36	36-Item Short Form Health Survey
TG	Triglycerides
TP	Total protein
UA	Uric acid
β2MG	Beta-2 microglobulin
LDL-C	Low-density lipoprotein cholesterol
